# La néphrotoxicite secondaire à l'hyperbilribinemie: à propos d'un cas

**DOI:** 10.11604/pamj.2015.22.313.8225

**Published:** 2015-11-27

**Authors:** Med Taieb Jomni, Mouna Largueche, Badreddine Ben Kaab, Imen Abdelaal, Syrine Bellakhal, Med Hédi Douggui

**Affiliations:** 1 Service de Médecine Interne, Hôpital des Forces de Sécurité Intérieures, La Marsa, Tunisie

**Keywords:** Hyperbilirubinemie, insuffisance renale, hypokaliémie, hyperbilirubinemia, renal failure, hypokalemia

## Abstract

L'hyperbilirubinémie à des taux élevés est nephrotoxique par divers mécanismes allant de la tubulopathie proximale aux dépôts massifs de bilirubine. Cette entité bien que décrite depuis le début du vingtième siècle est souvent méconnue dans la littérature moderne. Nous rapportons le cas d'un patient présentant une cirrhose probablement d'origine médicamenteuse (Amiodarone*), dont la fonction rénale s'est altérée parallèlement à la majoration de l'hyperbilirubinémie. Aucune autre cause de l'insuffisance rénale aigue n'a été retrouvée.

## Introduction

L'insuffisance rénale peut être associée à l'ictère. Elle est généralement secondaire à l'infection, l'hypovolémie, la nécrose tubulaire ou au syndrome hépato-rénal en cas de cirrhose. L'hyperbilirubinémie importante en elle-même peut occasionner une toxicité rénale. Néanmoins cette entité est souvent méconnue avec seulement quelques cas rapportés dans la littérature.

## Patient et observation

Nous rapportons l'observation d'un patient de 71 ans diabétique, hypertendu, ayant une fibrillation auriculaire paroxystique nécessitant sa mise sous Amiodarone*, admis pour exploration d'un ictère d'allure cholestatique évoluant depuis 3 mois et d'un seul tenant avec un prurit généralisé et anorexie sans saignement extériorisé. L'interrogatoire a éliminé une intoxication éthylique. L'examen clinique montre un ictère cutané franc avec des lésions de grattage, sans grosse vésicule biliaire palpable, ni ascite ni splénomégalie. Par ailleurs on ne retrouve pas d'hépatomégalie mais le bord inférieur du foie était tranchant. L'examen des urines aux bandelettes urinaires n'a pas montré de protéinurie ni d'hématurie. Les examens biologiques ont objectivéune anémie normochrome normocytaire, une hyperbilrubinémie avec un syndrome de cholestase, une hypoalbuminémie avec un bloc béta-gamma à l’électrophorèse des protides sériques, une hyperthyroïdie, sans altération de la fonction rénale ni troubles ioniques: Hémoglobine:12g/dl, leucocytes: 4400/mm^3^, les plaquettes: 176000/mm^3^, TP: 75%, Bilirubine totale: 100µmol/l, Bilirubine conjuguée: 60µmol/l, PAL: 291U/L, GGT: 269U/L, ASAT/ALAT: 59/39 U/L, Albumine: 26g/l, Gammaglobuline: 16g/l, CRP: 26mg/l, Créatinine: 53µmol/l, Natrémie: 135mmol/l, Kaliémie: 3,7mmol/l, FT4= 2,63pmol/l, TSH normale. L’échographie abdominale, le scanner thoraco-abdomino-pelvien et la cholangio-IRM ont conclu à un foie d'hépatopathie chronique, sans anomalies des voies biliaires ni lésion focale, avec une splénomégalie, sans autres anomalies.

La gastroscopie n'a pas retrouvé de signes endoscopiques d'hypertension portale. Dans le cadre du bilan étiologique de la cirrhose, les sérologies virales et le bilan immunologique étaient négatifs. L'origine médicamenteuse (Amiodarone*) étant probable, l'anti-arythmique a été arrêté et le patient a été mis sous bétabloquants. L’évolution était marquée sur le plan clinique par l'aggravation de l'ictère, sans autres signes associés, avec une majoration de l'hyperbilirubinémie (Bilirubine totale à 442µmol/l, Bilirubine conjuguée à 25µmol/l), l'apparition d'une insuffisance rénale aigue (créatinine à 250µmol/l, urée= 15mmol/l), avec une hyponatrémie à 133mmol/l, une hypokaliémie à 3mmol/l sans signes électriques, et une acidose métabolique (pH = 7.36 et bicarbonates = 14mmol/l). A l'ionogramme urinaire, on a objectivé une kaliurèse inadaptée à 40 mmol/l avec une natriurèse à 50 mmol/l. L’échographie rénale n'a pas retrouvé d'anomalies ainsi que l'analyse du sédiment urinaire. L'insuffisance rénale a persisté même après hydratation par voie intraveineuse et des perfusions d'albumine. L'hypokaliémie récidivait à l'arrêt de la supplémentation intra-veineuseen potassium. La ponction-biopsie rénale n'a pu être réalisée devant les anomalies de l'hémostase. Devant cette insuffisance rénale aigue avec une atteinte tubulaire, sans étiologie évidente, dont l'apparition était concomitante à une hyperbilirubinémie majeure, le diagnostic le plus probable est la néphropathie secondaire à l'hyperbiliribinémie.

## Discussion

La néphropathie secondaire à l'hyperbilirubinémie est une cause souvent méconnue d'insuffisance rénale [1]. Durant la dernière décennie, quelques cas seulement ont été rapportés [2, 3]. Le mécanisme physiopathologique n'est pas encore bien connu. L'atteinte rénale peut aller de la tubulopathie proximale aux lésions tubulaires extensives [1]. Cette atteinte serait secondaire à un effet toxique direct des constituants de la bile sur les cellules tubulaires et aux dépôts de bilirubine engendrant une obstruction tubulaire [4]. La ponction biopsie rénale quand elle est faite affirme le diagnostic en montrant des lésions épithéliales tubulaires avec une surcharge épithéliale pigmentaire et des dépôts de bilirubine qui peuvent obstruer les tubules [4]. Le risque de toxicité tubulaire est accru en cas d'hyperbilirubinémie majeure (surtout au-delà de 340umol/l). L'hypoalbuminémie et l'acidose favorisent aussi cet effet délétère par le biais de la diminution de la liaison de la bilirubine et des acides biliaires à l'albumine, ce qui permet leur filtration glomérulaire et augmente l'exposition des tubules [4]. Une récupération de la fonction rénale peut être obtenue en cas de contrôle précoce du taux de bilirubine [1, 5]. Par contre elle peut être retardée en présence de dépôts tubulaires extensifs de bile [1]. Notre patient avait une fonction rénale normale quand la bilirubinémie était de 100umol/l. Deux mois après, il a présenté une altération de sa fonction rénale concomitante à une majoration de l'hyperbilirubinémie qui a dépassé 400umol/l. Il avait par ailleurs une hypoalbuminémie et une acidose métabolique. Il n'y avait pas d'arguments en faveur d'une origine pré-rénale, post -rénale ou d'un syndrome hépato-rénal,et l'analyse des urines n'a pas montré d'anomalies en dehors d'une natriurèse et d'une kaliurèse inadaptées. La néphropathie secondaire à la bilirubine était donc le diagnostic le plus probable. Devant la persistance de l'ictère, la créatinémie est restée élevée.

## Conclusion

La néphropathie secondaire à l'hyperbilirubinémie est un diagnostic auquel il faut penser quand l'insuffisance rénale aigue coïncide avec des taux élevés de bilirubine plasmatique. La confirmation diagnostique est histologique et le traitement dépend de l'hépatopathie sous-jacente. La normalisation du taux de bilirubine est souvent suivie de la récupération de la fonction rénale.
